# Diversity of retromer-mediated vesicular trafficking pathways in plants

**DOI:** 10.3389/fpls.2023.1184047

**Published:** 2023-06-20

**Authors:** Suryatapa Ghosh Jha, Emily R. Larson

**Affiliations:** ^1^ William Myron Keck Science Department - Biology, Claremont McKenna, Pitzer, and Scripps Colleges, Claremont, CA, United States; ^2^ School of Biological Sciences, University of Bristol, Bristol, United Kingdom

**Keywords:** retromer, endomembrane system, retriever, vesicle traffic, protein recycling

## Abstract

The plant endomembrane system is organized and regulated by large gene families that encode proteins responsible for the spatiotemporal delivery and retrieval of cargo throughout the cell and to and from the plasma membrane. Many of these regulatory molecules form functional complexes like the SNAREs, exocyst, and retromer, which are required for the delivery, recycling, and degradation pathways of cellular components. The functions of these complexes are well conserved in eukaryotes, but the extreme expansion of the protein subunit families in plants suggests that plant cells require more regulatory specialization when compared with other eukaryotes. The retromer is associated with retrograde sorting and trafficking of protein cargo back towards the TGN and vacuole in plants, while in animals, there is new evidence that the VPS26C ortholog is associated with recycling or ‘retrieving’ proteins back to the PM from the endosomes. The human VPS26C was shown to rescue *vps26c* mutant phenotypes in *Arabidopsis thaliana*, suggesting that the retriever function could be conserved in plants. This switch from retromer to retriever function may be associated with core complexes that include the VPS26C subunit in plants, similar to what has been suggested in other eukaryotic systems. We review what is known about retromer function in light of recent findings on functional diversity and specialization of the retromer complex in plants.

## 1 Introduction

The endomembrane system is a highly coordinated network that controls cellular function and development in living organisms. This system is responsible for the synthesis, sorting, and distribution of proteins within cells in a manner that is often crucial for their growth and viability. The endosomal trafficking of cargo to the storage and lytic vacuoles in plants, or the lysosome in animal systems, involves the action of Soluble NSF Attachment Receptors (SNAREs) that control the fusion of vesicles carrying cargo to target membranes. A retrograde trafficking pathway that involves the retromer complex is necessary for transporting cargo and endosomal membrane proteins to the *trans*-Golgi network (TGN) and coordinated with the trafficking of cargo to the vacuole. In animals, retromer defects can impair insulin signaling (Ma et al., 2014; Yang et al., 2016), while mutations in the core retromer subunits are linked to neurological disorders, including Parkinson’s (Williams et al., 2017) and Alzheimer’s diseases (Tammineni et al., 2017). In plants, a retromer defect can result in altered growth and development and impair the ability of the plant to adapt to stress. For example, in *Arabidopsis thaliana*, retromer loss of function is linked to defects in root hair growth and patterning, shoot gravitropism, increased sensitivity to drought stress, and immunity-associated cell death pathways (Munch et al., 2015; Jha et al., 2018; Liang et al., 2022). The coordination of endosomal trafficking pathways between the TGN and the vacuole was also underscored by reports that a genetic interaction between specific retromer-dependent endosomal trafficking pathways and a VTI-SNARE dependent pathway to the lytic vacuole are required for both shoot gravitropism (Hashiguchi et al., 2010) and root hair growth in Arabidopsis (Jha et al., 2018).

The trafficking of receptor proteins from late endosomes and Golgi/TGN to the vacuole is facilitated by the retromer complex. Retromers function by binding to specific receptors on transport vesicles and directing them back to the TGN for further sorting and distribution. However, the identity of the protein complexes involved in retrograde endosomal trafficking in eukaryotes has expanded to include a retromer-like “retriever” complex (McNally et al., 2017). This complex is formed by interactions between core retromer-like proteins that traffic cargo from the endosomes to the plasma membrane rather than the TGN (McNally et al., 2017). While it is clear that the retromer functions between plants and animals are not always directly comparable, studies in multiple systems have suggested that the core subunits of the retromer complex could be responsible for specializing its subcellular localization and function (McNally et al., 2017; Jha et al., 2018; Ivanov and Robinson, 2020; Zelazny et al., 2013b).

In this review, we summarize our understanding of the retromer complex in plants, focusing on a newly described, evolutionarily conserved Vacuolar Protein Sorting (VPS) 26 protein (VPS26C) that is required for root hair growth and has a putative retriever function similar to its human ortholog, DSCR3/VPS26C. We also discuss genetic studies that implicate interactions between trafficking pathways regulated by the retromer and the VTI family of SNAREs in controlling plant development in plants. Finally, we discuss the recent advances in retromer biology, the current gaps in knowledge, and the tools we need to further elucidate the functional scope of retromer/retriever-dependent endosomal recycling pathways in plant development.

## 2 The classic retromer complex

The retromer complex was initially characterized in yeast and shown to function in the endosome-to-Golgi trafficking of the transmembrane receptor for vacuolar carboxypeptidase Y, Vps10p (Seaman et al., 1998). Retromers were subsequently defined as a hetero-pentameric complex of large and small subunits. In yeast, the large retromer subunit consists of three highly conserved proteins, VPS35, VPS29, and VPS26 (Haft et al., 2000; Seaman, 2004), that is responsible for interacting with cargo proteins in a retrograde trafficking pathway from endosomes to the TGN (Seaman et al., 1998; Burda et al., 2002). The small subunit of the retromer in yeast is a dimer of two nexin proteins, VPS5p, and VPS17p that function in membrane binding, curvature, and tubulation (Horazdovsky et al., 1997; Carlton et al., 2005).

In mammalian systems, the large retromer subunit is composed of VPS35 and VPS29 proteins that complex with one of two VPS26 paralogs, VPS26A or VPS26B (Seaman et al., 1998). VPS26A and VPS26B in mice are not functionally redundant (Bugarcic et al., 2011), suggesting that different versions of the large retromer complex function in distinct endosomal trafficking pathways. The small subunit of the mammalian retromer is a heterodimer of two sorting nexins, either SNX1 or SNX2 and SNX5 or SNX6, which function in binding membranes and recruiting the large retromer subunit to the endosomal membrane (Bonifacino and Hurley, 2008; Collins, 2008). While the large and small retromer subunits form a stable complex in yeast, the interaction between these two subunits is much weaker and transient in other eukaryotes (Harbour and Seaman, 2011; Swarbrick et al., 2011). Along with the sorting nexins of the small retromer subunit, many animal genomes encode additional sorting nexins that function in retrograde trafficking pathways that involve the retromer complex. For example, SNX3 can interact with the large retromer subunit to control endosomal trafficking pathways involved in sorting the transcription factor Wnt (Harterink et al., 2011), while SNX27 is a cargo adapter for the large retromer subunit on endosomal membranes (Gallon et al., 2014). The Wiscott-Aldrich syndrome and SCAR Homolog (WASH) complex also associates with the retromer subunits and nexins to coordinate actin nucleation and control endosomal sorting of transmembrane receptors (Bartuzi et al., 2016).

The retromer in animal systems can retrieve and recycle transmembrane receptors from endosomal membranes to and from the plasma membrane and the TGN, respectively, and mediate transport between organelles (**Table 1**). The retromer-mediated retrieval of receptors includes Carboxypeptidase Y (Seaman et al., 1998), Bone Morphogenetic Protein (BMP) Type I receptor SMA-6 (Gleason et al., 2014), the phagocytic receptor CED-1 from phagosomes to the plasma membrane (Chen et al., 2010), and neurotransmitter receptor, GLR-1, from dendrites to the cell body (Zhang et al., 2012) in *Caenorhabditis elegans*. Retromer-mediated recycling of G-Protein Coupled Receptors (GPCRs) have also been reported in studies using human cell lines (Bugarcic et al., 2011; Temkin et al., 2011) and *Drosophila melanogaster* (Wang et al., 2014). In addition, retromer complexes have been implicated in the retrograde trafficking of transmembrane proteins from the plasma membrane to the TGN in *C. elegans* (Bai and Grant, 2015), and transport between peroxisomes and mitochondria in human cell lines (Braschi et al., 2010), all of which highlight the essential role of the retromer complex in regulating signaling pathways and organelle transport that are crucial for cell function and development.

**Table 1:** List of receptors trafficked by retromer-dependent pathways in non-plant systems.

Study system	Receptor	Trafficking Route	Reference
*Caenorhabditis elegans*	Carboxypeptidase Y	Endosomes to TGN	Seaman et al., 1998
*Caenorhabditis elegans*	BMP Type I receptor SMA-6	Endosomes to TGN	Gleason et al., 2014
*Caenorhabditis elegans*	Phagocytic receptor CED-1	Phagosomes to PM	Chen et al., 2010
*Caenorhabditis elegans*	Neurotransmitter receptor GLR-1	Dendrites to cell body	Zhang et al., 2012
Human cell lines	G-Protein-Coupled Receptors (GCPRs)	Endosomes to TGN	Bugarcic et al., 2011; Temkin et al., 2011
*Drosophila melanogaster*	G-Protein-Coupled Receptors (GPCRs)	Endosomes to TGN	Wang et al., 2014
Human cell lines	Mitochondria Anchored Protein Ligase (MAPL)	Mitochondria to Peroxisome	Braschi et al., 2010

A study using human cell lines demonstrated the formation of the retromer-like ‘retriever’ complex, involving the DSCR3/VPS26C protein (McNally et al., 2017). Using biochemical assays, this study demonstrated that DSCR3/VPS26C forms a complex with VPS29 and a VPS35-like (C16orf62) protein, leading to three major conclusions about the retriever complex in human cells. Firstly, the retriever complex localizes to endosomal membranes through its recruitment by the Copper Metabolism MURR1 Domain (COMMD)/Coiled-Coil Domain Containing CCDC22/CCDC93 (CCC) complex, which associates with the WASH complex to mediate transmembrane receptor sorting (Bartuzi et al., 2016). Secondly, DSCR3/VPS26C mediates the interaction between the retriever complex and the cargo adaptor Sorting Nexin (SNX) 17 to regulate the retrograde trafficking of a subset of plasma membrane proteins. Lastly, siRNA suppression of CCDC22 and CCDC93 interrupted the endosomal association of the retriever complex with SNX17, which led to the mis-sorting of the α_5_β_1_ integrin, one of the VPS26C/SNX17 cargo proteins.

**Figure 1:**
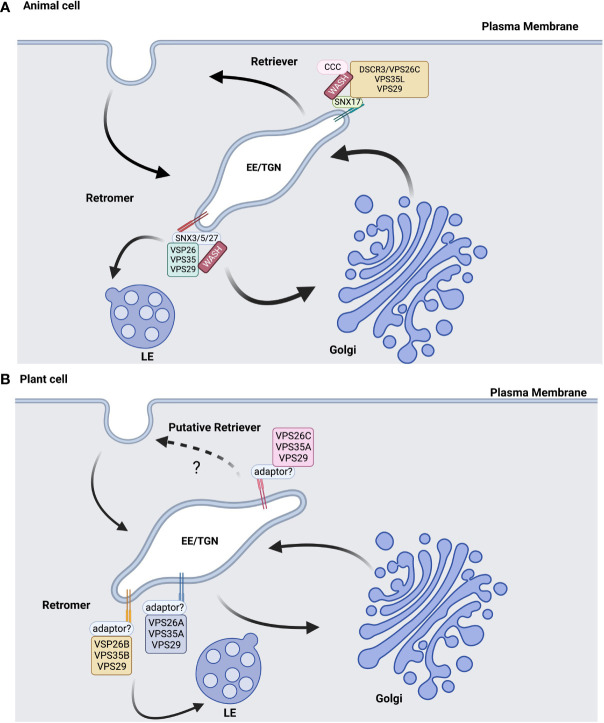
Retromer/retriever models in animal and plant cells are similar but not identical. **(A)** In animal cells, the retromer complex is composed of VPS25, VPS35, and VPS29 core subunits, syntaxin (SNX) small subunits, and the WASH complex in trans-Golgi Network (TGN)-derived early endosomes (EE). Different SNX small subunits and the CCC complex associate with WASH to recruit the retriever cargo back to the plasma membrane and differentiate this complex from the retromer, which sorts cargo for retrograde trafficking towards the Golgi and late endosomes (LE). **(B)** In plants, the core subunits may determine the retromer and retriever functions, with VPS26C potentially contributing to returning endomembrane cargo back to the PM as part of a retriever-like complex. Diagrams informed by the studies referenced in this review and were created with BioRender.com.

This working model was used to characterize the retromer-dependent trafficking that facilitates membrane curvature, tubulation, and retrograde transport of cargo in animal systems. However, this model was challenged by that of the retrograde transport of the CI-MPR receptor from late endosomal membranes to the TGN (Kvainickas et al., 2017; Simonetti et al., 2017). There is new evidence that SNX proteins within the small retromer subunit are required for retrograde trafficking of the CI-MPR receptor in humans, while downregulation of the VPS35 large retromer subunit had no effect on the recycling of this receptor from late endosomal membranes (Kvainickas et al., 2017; Simonetti et al., 2017). Hence, there is support for a new model for retromer trafficking in which the small subunit is essential for the retrograde trafficking of receptors from late endosomal membranes to the TGN (**Figure 1A**).

## 3 Retromer function in plants

The availability of several plant genomes and the strong conservation of retromer subunit sequences were used to identify retromer subunits in a variety of plant species. In Arabidopsis, VPS35 and VPS26 are both encoded by three gene family members, while VPS29 is a single copy gene. In contrast, the small retromer subunit is composed of a heterodimer containing SNX1 and either SNX2a or SNX2b, which are homologs of the yeast VPS5p sorting nexin (Niemes et al., 2010). In plants, genetic studies showed that the core retromer functions independently of its interaction with the sorting nexins (Pourcher et al., 2010), which is a diversion from mammalian systems where the retromer complex assembly involves sorting nexins (Wassmer et al., 2009; Zelazny et al., 2013a) (**Figure 1B**).

The large and small retromer subunits are required for endosomal trafficking pathways that control diverse aspects of plant development. Genetic analysis has shown that single *vps26a* and *vps26b* mutants have no developmental defects in Arabidopsis, but the *vps26a vps26b* double mutant displays severely stunted growth, indicating functional redundancy between the VPS26 paralogs (Zelazny et al., 2013b). A similar analysis of VPS35 family members showed that a *vps35b vps35c* double mutant exhibits a dwarfed growth phenotype, early senescence, and defects in trafficking proteins to the storage vacuole (Yamazaki et al., 2008). Mutations in *vps29* and *snx1* have similar developmental phenotypes and defects in organ initiation as that observed in *pin* mutants. These results suggest that VPS29 and SNX1 function in a common pathway to regulate PIN-FORMED (PIN) protein recycling and cell polarity (Jaillais et al., 2007; Kleine-Vehn et al., 2008). In Arabidopsis, retromer subunit mutants describe a role for VPS35A and VPS26A in shoot gravitropism (Hashiguchi et al., 2010), VPS35B and VPS26B in innate immunity (Munch et al., 2015), VPS29 in the transport of Sugar-Dependent-1 (SDP1) from peroxisomes to oil bodies during seedling development (Thazar-Poulot et al., 2015), and SNX1 in the trafficking of IRT1 and the efficiency of iron uptake machinery in roots (Ivanov et al., 2014). The retromer complex was also implicated in the delivery of lipid enzymes to viral replication organelles (VROs) in Tomato Bushy Stunt Virus (TBSV), which is a Tombusvirus in plants (Feng et al., 2021). These results highlight the diverse roles for both the large and small retromer subunits in endosomal trafficking pathways that control plant development.

Recent research on retromer biology has elucidated the mechanism of retromer recruitment processes in Arabidopsis. The plant specific endosomal regulator, BLISTER, was identified and characterized to function in membrane recruitment of the retromer core complex in the sorting and trafficking of soluble vacuolar proteins to the TGN and recycling of endocytosed plasma membrane proteins (Li et al., 2023). This report not only identified a mechanism of core retromer function but also retromer function in alternative trafficking pathways other than the conventional endosome to TGN pathways. Moreover, the ESCRT-associated protein ALIX is a conserved protein previously characterized to function in vacuolar degradation of abscisic acid receptors that also interacts with the retromer complex. In the absence of ALIX, the aberrant recruitment of VPS29 and VPS26 to the membrane results in defective localization and trafficking of Vacuolar Sorting Receptors (VSRs) (Hu et al., 2022). Additionally, retromer association with the gibberellic acid-signaling mediator DELLA mediates recycling pathways (Salanenka et al., 2018) and the interaction of the core retromer protein VPS35 with RabG3f to control late endosome-vacuole fusion (Rodriguez-Furlan et al., 2019; Zelazny et al., 2013b) reinforce the functional range of the retromer complex in regulating recycling pathways to the TGN and vacuole, as well as to the plasma membrane.

### 3.1 Evolutionary divergence of VPS26 function in plants and other eukaryotes

Phylogenetic analysis of genes encoding the VPS26 large retromer protein has shown that DSCR3/VPS26C represents a third VPS26 gene family member that is evolutionarily conserved and part of a smaller, monophyletic clade distinct from the *VPS26A* and *VPS26B* homologs across plant and animal species (Koumandou et al., 2011). Along with VPS26C, other subunits of the retriever complex are very well conserved and potentially co-evolved from the last eukaryotic ancester, being lost only in fungi (McNally et al., 2017). This strong conservation of retromer/retriever genes in eukaryotes indicates the molecular functions are crucial for most all eukaryotic cell function. In Arabidopsis, VPS26C forms a complex with VPS35A and VPS29 that is required for the regulation of polarized growth in root hairs. VPS26C orthologs exhibit functional conservation, indicated by the complementation of the *vps26c* mutant phenotype in Arabidopsis by the human *DSCR3/VPS26C* (Jha et al., 2018). Like the Arabidopsis VPS26C subunit, the DSCR3/VPS26C human ortholog also associates with VPS29 and a VPS35-like protein (McNally et al., 2017). Moreover, consistent with Zelazny et al. (2013b), who suggested that the localization of the retromer complexes could be mediated by the VPS26 isoforms, VPS26C did not localize to wortmannin-sensitive compartments, which indicated that it does not require RabG3f for its recruitment (Jha et al., 2018).

Differences in retromer and retriever function are mediated in part by the cargo proteins that they traffic to the TGN or plasma membrane, as well as by the cargo adaptor used to recruit these complexes to the endosomal membrane. Proteomic and genetic analyses of the VPS26C complex in human cell culture reported that the VPS26C retriever complex participates in a trafficking pathway directed to plasma membrane (McNally et al., 2017), a deviation from that of the retromers that are conventionally involved in trafficking cargo to the Golgi/TGN. In addition, the functional conservation of VPS26C orthologs in different organisms could indicate that VPS26C evolved a new functionality in endosomal trafficking pathways that are distinct from the classic retromer complex in Arabidopsis. The membrane recruitment of VPS26C in mammalian cells involved the CCDC22/CCDC93/COMMD protein complex. Arabidopsis has homologs of CCDC22 and CCDC93 but not COMMD (Reviewed in Law et al., 2022). Therefore, further investigation will be needed to determine the cargo and proteins involved in the membrane recruitment of the VPS26C complex and the functional specificity of the VPS26C-dependent complexes in plants (**Figure 1**).

### 3.2 Genetic crosstalk between retromers and the VTI SNARE family

The VTI SNARE family participates in trafficking cargo to the lytic or storage vacuoles in Arabidopsis (Sanmartin et al., 2007; Larson et al., 2014). Null mutants for each of the VTI SNAREs exhibit unique phenotypes, suggesting that they have distinct functions in plants. The *vti11* mutant has defective leaf vasculature (Shirakawa et al., 2009), aberrant central lytic vacuole formation (Sanmartin et al., 2007), and a shoot agravitropic phenotype, while those of *VTI12* display no developmental phenotype when grown on a nutrient-rich medium but have accelerated senescence when grown on nutrient-deficient media, suggesting a role in plant autophagy (Surpin et al., 2003). Moreover, *vti12* plants also exhibit abnormal accumulation of 12S globulin precursors in siliques, indicating VTI12-mediated trafficking of vacuolar storage proteins (Sanmartin et al., 2007); while *vti13* is defective in root hair growth and cell wall organization in root epidermal and hair cells in Arabidopsis, suggesting these trafficking pathways are required for cell shape and growth (Larson et al., 2014).

Studies have linked VTI11 and VTI13-mediated anterograde trafficking pathways to the lytic vacuole with endosomal trafficking pathways mediated by the retromer. A suppressor screen of *vti11* found that mutations within genes encoding the core retromer proteins VPS35A and VPS26A were sufficient to suppress the *vti11* shoot agravitropic ‘zigzag’ phenotype in double mutants (Hashiguchi et al., 2010). Although the mechanism of this genetic interaction is not defined, the mis-sorting of membranes to vacuoles in *vti11 vps26a* or *vti11 vps35a* double mutants resulted in a recovery of the vacuolar dynamics necessary for amyloplast movement in endodermal cells that restored shoot gravitropism (Hashiguchi et al., 2010). A similar interaction between mutations in the VPS26C retromer subunit and the VTI13 SNARE was described by (Jha et al., 2018), where a loss-of-function mutation for *VPS26C* restored the root hair growth and wall organization phenotype of the *vti13* mutant. While the cellular mechanism responsible for this suppression is currently unknown, it is interesting that VPS35A physically interacts both with VPS26A and VPS26C in Arabidopsis (Jha et al., 2018; Zelazny et al., 2013b) and that VPS35B and VPS35C cannot substitute for VPS35A in endosomal trafficking pathways that control shoot gravitropism (Hashiguchi et al., 2010). VPS35A function is also required for cargo trafficking to the lytic vacuole (Nodzyński et al., 2013), indicating that retromer function may be required for multiple trafficking pathways between both the lytic vacuole and TGN. Understanding the coordination of the endosomal trafficking pathways that are required for trafficking of cargo between these organelles and the identity of the proteins involved in these pathways is required to determine the cellular mechanisms that mediate the diverse developmental processes in plants.

## 4 Conclusion

In plants, the current literature addresses the interactions of the core retromer subunits and the effects these proteins have on developmental pathways. These studies characterized retromer function using genetics-based approaches, including loss-of-function mutants to correlate retromer function with developmental defects. Although these studies are seminal in demonstrating the importance of retromer proteins, there is still a substantial gap in our knowledge about the cell biology of plant retromers, including the mechanism of their membrane recruitment, their potential interacting/adapter proteins, and the identification of their cargo. Studies have explored retromer mechanisms, but these studies are underrepresented in the existing literature. There are several key questions that need to be addressed to fill these gaps, some of which are outlined below:

1. VPS26C interacts with core retromer subunit VPS35A and VPS29. However, the human ortholog DSCR3/VPS26C is part of a ‘retriever’ complex that recycles cargo to the plasma membrane. The functional conservation of VPS26C orthologs in humans and plants begs the question: Does the plant VPS26C function in retromer (endosome to TGN) or retriever (endosomes to plasma membrane) pathways?
2. Is the membrane recruitment process of retromers and retrievers the same in plants and animals?
3. Given that VPS26C interacts with core retromer subunits that function in the classical retromer pathway, is the retromer/retriever distinction in plants cell type-dependent?
4. If there are diverse recycling routes, there must be diverse types of cargo. How could we identify these cargoes?

Using and developing new tools in immunochemistry, proximity-based labeling and proteomics, and high-resolution imaging could help address these gaps in retromer cell biology by enabling the investigation of subcellular phenotypes and interactomes of retromers, identifying cargo and define the diverse pathways they function in to maintain optimal plant development.

## Author contributions

SGJ and ERL equally contributed to the conception and writing of this review. All authors contributed to the article and approved the submitted version.
